# Rheumatic Manifestations in Patients with Idiopathic Inflammatory Bowel Disease: A Single Tertiary Centre, Interdisciplinary Study

**DOI:** 10.31138/mjr.291123.ept

**Published:** 2025-05-23

**Authors:** Athanasios Kavvadias, Maria Karavasili, Eleftherios Pelechas, Maria Veatriki Christodoulou, Voulgari Paraskevi V., Konstantinos H. Katsanos, Dimitrios K. Christodoulou

**Affiliations:** 1 Department of Gastroenterology;; 2 Department of Ophthalmology;; 3 Department of Rheumatology; and; 4 Department of Radiology, Medical School and University Hospital of Ioannina, Greece

**Keywords:** rheumatological manifestations, enthesitis, inflammatory bowel disease, musculoskeletal ultrasound

## Abstract

**Background:**

Inflammatory bowel disease is closely associated with extraintestinal manifestations. Among them, joint involvement and enthesitis are the most frequent resembling a spondyloarthropathy. Enthesitis may be clinically silent in a high proportion of these patients without any clinical signs or a diagnosis of spondyloarthritis.

**Objectives:**

To evaluate, with the use of musculo-skeletal ultrasound, the frequency and location of musculoskeletal manifestations in patients with inflammatory bowel disease.

**Methods:**

Fifty patients with a definite diagnosis of inflammatory bowel disease were prospectively recruited and clinically evaluated by a rheumatologist for relevant musculoskeletal symptoms. All of the patients underwent a thorough musculo-skeletal ultrasound examination of both knees, patellae, as well as upper and lower limbs, in order to identify synovitis, and enthesitis. Musculo-skeletal ultrasound examination was performed at 12 entheses. The ultrasound abnormalities were scored according to the Madrid Ankylosing Spondylitis Enthesitis Index. Results: The musculo-skeletal ultrasound examination performed, revealed the presence of synovitis in 24% and enthesitis in 64% of these patients. More specifically, in 6.3% of the “Quadriceps tendon”, in 40.6% in the “Achilles tendon”, in 40.6% in the “Quadriceps and Achilles”, in 9.4% in the “Triceps, Quadriceps and Achilles’ and at a rate of 3.1% in the ‘Patella’.

**Conclusions:**

The musculo-skeletal ultrasound verified that a significant percentage of the patients exhibit some type of “musculoskeletal manifestations”, with enthesitis to be the most common of them in patients suffering from inflammatory bowel disease.

## INTRODUCTION

Inflammatory bowel diseases (IBD), which encompass Crohn’s Disease (CD) and ulcerative colitis (UC), are inflammatory conditions of unknown origin that affect the gastrointestinal tract, impacting nearly four million people globally.^1^ Extraintestinal manifestations are common in IBD patients and present with a variety of clinical patterns. Multiple organs, such as the skin, joints, and eyes, can be affected simultaneously, suggesting a possible genetic predisposition.^2^ Joint involvement is the most frequent extraintestinal manifestation, affecting 16–33% of IBD patients depending on diagnostic criteria and patient selection.^3^ Spondyloarthropathies (SpA), which include various forms of inflammatory arthritis, can involve both the spine and peripheral joints. Axial and peripheral arthritis may appear before the diagnosis of IBD and have a similar prevalence in both CD and UC.^4^ Additionally, SpA is associated with macroscopic (endoscopic) gastrointestinal inflammation (GI) in 30–44% of cases^5^ and microscopic (histologic) inflammation in 46–66% of cases.^6^ The clinical presentation of musculoskeletal involvement in IBD patients varies widely and can present as either articular (arthritis) or periarticular inflammation including enthesitis, myositis, or soft tissue rheumatism (fibromyalgia), ranging from transient and mild symptoms to persistent and disabling ones^7^. This is a significant concern as it increases disability and deteriorates the quality of life for those affected. Articular symptoms are believed to align with the clinical progression of IBD,^8^ but there is no clear data depicting this correlation. Furthermore, there is a growing body of evidence supporting that IBD-related arthritis is the type of SpA that occurs in patients affected by IBD, with an incidence up to 50% during the IBD course.^9^ However, their prevalence is often underestimated due to the transient nature of certain oligoarticular patterns, the use of chronic corticosteroid treatment,^10^ or the misattribution of enthesitis due to overuse.^11^ Although patients with IBD and SpA share numerous clinical, immunologic, and genetic characteristics, the precise relationship between these two conditions has not been fully established. Therefore, a multidisciplinary approach is essential to ensure early detection and appropriate treatment, preventing poor outcomes. Musculoskeletal ultrasonography (MSUS) has proven to be an effective tool for early detection of joint and tendon involvement in patients with various rheumatic conditions, capable of identifying pathological changes even in the absence of symptoms.^12–17^

The aim of this study was to use MSUS to evaluate the frequency and location of musculoskeletal manifestations in patients with IBD.

## METHODS

Fifty patients with a confirmed diagnosis of IBD were enrolled in this study. The diagnosis was established based on clinical, histological, endoscopic, radiological, and laboratory data by an experienced gastroenterologist. This prospective study was conducted through the collaboration of the outpatient gastroenterology and rheumatology departments at the University Hospital of Ioannina in Greece, spanning from January 2018 to January 2019. Participants were clinically assessed by a rheumatologist for musculoskeletal symptoms and signs, such as arthralgias, peripheral arthritis, and axial involvement, including the sacroiliac joints. All patients also underwent a comprehensive MSUS examination. More specifically, the wrist joints, metacarpophalangeal, proximal interphalangeal and distal interphalangeal joints of the hands, as well as both knees have been investigated for the presence of grey scale synovitis and synovial power-Doppler signal, in order to identify synovitis^18–19^ whereas the knees, patellae, upper and lower limbs, in order to identify enthesitis.^20^ All the aforementioned anatomical points have been evaluated using the Madrid Sonographic Enthesitis Index (MASEI) for the possibility of subclinical inflammation,^21^ and ultrasound synovitis was identified according to the OMERACT definitions.^22^ Specifically, six compression sites were evaluated: the plantar fascia, Achilles tendon, distal and proximal patellar ligaments, distal quadriceps, and brachialis triceps tendon. The MASEI score, a weighted score previously calculated through logistic regression, estimates the severity of three elemental lesions: calcifications (scored 0–3), power Doppler signal (scored 0–3), and erosions (scored 0–3), along with scoring tendon structure, tendon thickness, and bursae (scored 0–1). Calcifications were scored as follows: 0 if not present, 1 if a small calcification or an ossification with irregular enthesis cortical bone profile was observed, 2 if there were evident enthesophytes (hyperechoic spurs forming at tendon insertions into bone in the direction of the tendon pull) or medium-sized calcifications or ossifications, and 3 if large calcifications or ossifications were present. The total MASEI score was the sum of the scores for both sides (12 entheses).^23^

The MSUS equipment used for the study is the Esaote MyLab™Twice with a linear probe LA533 Appleprobe 3.0/13.0 MHz. The exclusion criteria for the study were as follows: age under 18 years, a history of any infectious, degenerative, crystal, or inflammatory arthritis, musculoskeletal disease, psoriasis, lower limb peripheral neuropathy, uveitis, a history of knee or ankle surgery, or corticosteroid injections in the examined structures. All participants provided informed consent to participate in the study, which was conducted in accordance with the Declaration of Helsinki.^24–25^

## RESULTS

Fifty patients with IBD were recruited for this study (25 with CD, and 25 with UC), 28 male and 22 female. **Table 1** shows the characteristics of the IBD patients.

Patients with IBD in this study were receiving anti-TNF agents in a percentage of 70% (Infliximab 60%, Adalimumab 10%) and integrin receptor antagonists (Vedolizumab) in a percentage of 18%. Immunosuppressive drugs (Azathioprine) were receiving 20% of the patients (10% as monotherapy and 10% in co-administration with infliximab). Furthermore, the patients with UC were under treatment with aminosalicylates (Mesalazine) in a percentage of 40%.

**Table 1.** Patient characteristics.

		%	N
Sex	M	56%	28
	F	44%	22
IBD	Ulcerative Colitis	50%	25
	Crohn’s Disease	50%	25
Age	20 – 40 years	28%	14
	41 – 60 years	50%	25
	> 61 years	22%	11
Age characteristics	UC (mean - S/D – Min - Max)	52,6 / 15,0 / 27 / 85			
	CD (mean - S/D – Min - Max)	45,2 / 14,3 / 21 / 72			
Smoking habit	Yes	70%	35
	No	30%	15
Physical activity^28^	No	24%	12
	Light	32%	16
	Moderate	38%	19
	Vigorous	6%	3
Treatment	Infliximab	60%	30
	Adalimumab	10%	5
	Vedolizumab	18%	9
	Azathioprine	20%	10
	Mesalazine (UC patients)	40%	10
Disease duration	UC (mean, S/D, Min, Max)	15,8 / 11,2 / 2 / 42			
	CD (mean, S/D, Min, Max)	12,9 / 9,0 / 1 / 31			

M: male; F: female; IBD: inflammatory bowel disease; UC: ulcerative colitis; CD: Crohn’s disease; %: percentage of patients; N: absolute number of patients; S/D: standard deviation; Min: minimum; Max: maximum.

Of the patients asked, 52% reported arthralgias while on examination, 90% of these patients had signs of peripheral arthritis and 10% of axial involvement (64% of them were patients with CD and 36% with UC).

The MSUS examination of all patients revealed the presence of synovitis in 24% of them and enthesitis in 64% (**Table 2**)**.**

**Table 2.** Ultrasound findings.

		%	N
**Synovitis**	Yes	24%	12
	No	76%	38
**Enthesitis (64%, N32)**	Distal quadriceps tendon	6,3%	2
	Achilles tendon	40,6%	13
	Distal quadriceps and	40,6%	13
	Achilles tendon		
	Distal quadriceps, brachialis triceps, and Achilles tendon	9,4%	3
	Distal and proximal patellar ligaments	3,1%	1
**MASEI index (score)**	UC (mean - S/D – Min - Max)	3,4 / 2,4 / 0 / 9			
	CD (mean - S/D – Min - Max)	3,3 / 2,7 / 0 / 10			

CD: Crohn’s disease; UC: ulcerative colitis; S/D: standard deviation; Min: minimum; Max: maximum.

More specifically, the distribution of enthesitis locations was as follows: 6,3% in the quadriceps tendon, 40.6% solely in the Achilles tendon, 40.6% in both the quadriceps and Achilles tendons, while 9.4% in the triceps, quadriceps, and Achilles tendons, and 3.1% in the patella.

## DISCUSSION

The present study investigated the prevalence of musculoskeletal manifestations in patients with IBD.

The current results showed that more than half of the study population (52%) had some kind of musculoskeletal manifestations. These results seem to be in accordance with the prospective study made by Hammoudeh and colleagues in 2018,^29^ where 127 patients with IBD were enrolled and the results showed that 57.5% of the population of the study had some kind of rheumatic event, while another study conducted by Al-Jarallah et al. in 2013 in Kuwait,^30^ reported UC and CD prevalence of musculoskeletal manifestations of 34.6% and 65.4%, respectively, with similar findings in the study by Isene et al. in 2015.^31^ Also, these results are confirmed by our study, 64% of the patients with musculoskeletal manifestations had CD and 36% UC. In addition, our study confirmed the results of the systematic literature review by Sakellariou et al. in 2022^32^ which concluded that among the tested structures with MSUS, entheseal involvement emerged as the most frequent finding. On the other hand, there are also studies pointing out that enthesitis is significantly more frequent in the UC group than the CD group (37,7% versus 25%) such as the study by Bertolini et al.^33^ Even if the results of this study are not in accordance with most studies regarding the UC/CD ratio of enthesitis, it is one study that identified and differentiated between acute and chronic entheseal lesions. Patients with longer disease durations showed a higher frequency of entheseal abnormalities on ultrasound assessment. Abnormal findings included entheseal thickening, hypoechogenicity, bony erosions, enthesophytes, and bursal enlargement. Entheseal thickening, hypoechogenicity, and bursal enlargement were classified as acute lesions, while bony erosions, calcifications, and enthesophytes were categorised as chronic lesions.^33^ In a recent review by Akrapovic et al. (2024), several interesting studies have been included and presented with some of them having conflicting results but every single study presenting an important point.^34^ Cantini et al. demonstrated that the frequency of enthesitis was significantly higher in IBD-SpA patients with accompanying psoriasis, while the prevalence of enthesitis did not differ between patients with UC and those with CD,^35^ a point we didn’t examine as psoriasis was described in our exclusion criteria. Husic et al. found no significant correlation between disease duration or IBD activity and MASEI score, but ultrasound-confirmed enthesitis was more common in IBD patients compared to healthy controls. There was no association between clinical IBD activity and MASEI or clinical IBD activity and erosion.^36^ Bandinelli et al suggested that enthesitis can also occur in early IBD, with its occurrence being unrelated to disease duration or activity and found that 16% of their patients showed a positive power-doppler signal at entheses without symptoms.^37^ In this review, the authors enclosed also the study from Hsiao et al who found that none of their subjects had peripheral joint pain or swelling, and physical examinations were unremarkable. However, despite these findings, positive ultrasound results were detected in 13 of the 14 patients.^38^

All the aforementioned studies, show that MSUS has been proved to be more sensitive than clinical examination in identifying synovitis, and more specific than clinical examination in identifying entheseal involvement.^39^

## CONCLUSION

In conclusion, a significant percentage of the sample exhibits some type of rheumatological manifestations, mainly in peripheral locations, that could affect the daily quality of life of those patients. Entheseal involvement emerged as the most sensitive finding. On the other hand, the maximum value of the MASEI score in UC patients was 9 and in CD patients 10, which did not meet the cutoff value of 18 in order to distinguish those patients as having a real spondyloarthropathy.^40^ In addition, there was no treatment change after the MSUS findings. The MSUS has gained increasing success due to the technical advances and the availability in an out-patient setting allowing an immediate application of the results to patient management, the low cost and good acceptability by the patients because it is a radiation-free tool.^41^ For these reasons, ultrasonography has been considered an interesting imaging technique to evaluate patients at higher risk of developing arthritis as IBD patients, but in our study, there was no need of changing the treatment plans. This could be explained by the strict exclusion criteria that were applied in the current study. Enthesitis should be evaluated using both physical examination and ultrasound as the reference standard. However, data on such assessments are currently limited, and future studies with more comprehensive cohorts are needed to better understand enthesitis. From our study but also from the literature there is no clear answer to whether enthesitis is an indicative sign of IBD activity, but the fact is that in most studies enthesitis is a prevalent finding in these patients. Due to the fact that enthesitis may be silent and asymptomatic, most patients are often not examined. Therefore, the cooperation between the gastroenterologist and the rheumatologist for the IBD patients is indispensable in order to recognise and timely treat them accordingly.

## AUTHORS‘ DISCLAIMER

No part of the revised manuscript contain copied passages, and no part oft he revised manuscript is published or posted elsewhere.

## AUTHOR CONTRIBUTIONS

AK: conception, acquisition, analysis, drafting, final approval
MK: acquisition, reviewing, final approval
EP: drafting, reviewing, final approval
MVC: analysis, acquisition, final approval
PVV: acquisition, critical review of data, final approval
KHK: interpretation of data, reviewing, final approval
DKC: acquisition, critical review of data, final approval All authors are responsible for the accuracy and the integrity of the present work.

## CONFLICT OF INTEREST

The authors declare that the research was conducted in the absence of any commercial or financial relationships that could result as a potential conflict of interest.
